# Factors associated with drug use in prison – results from the Norwegian offender mental health and addiction (NorMA) study

**DOI:** 10.1186/s40352-020-00112-8

**Published:** 2020-05-12

**Authors:** Anne Bukten, Ingunn Olea Lund, Stuart A. Kinner, Eline Borger Rognli, Ingrid Amalia Havnes, Ashley Elizabeth Muller, Marianne Riksheim Stavseth

**Affiliations:** 1 Norwegian Centre for Addiction Research, University of Oslo, Kirkveien 166, 0407 Oslo, Norway; 2 Section for Clinical Addiction Research, Oslo University Hospital, Oslo, Norway; 3 Norwegian Institute of Public Health, Oslo, Norway; 4 Justice Health Unit, Centre for Health Equity, Melbourne School of Population and Public Health, University of Melbourne, Melbourne, Australia; 5 Centre for Adolescent Health, Murdoch Children’s Research Institute, Melbourne, Australia; 6 School of Population and Public Health, University of British Columbia, Vancouver, Canada; 7 Griffith Criminology Institute, Griffith University, Brisbane, Australia; 8 Mater Research Institute-UQ, University of Queensland, Brisbane, Australia; 9 National Advisory Unit on SUD treatment, Oslo University Hospital, Oslo, Norway

**Keywords:** Prisoners, Drug use, Substance use disorders, Harm reduction, Treatment

## Abstract

**Background:**

Remarkably little is known about drug use during imprisonment, including whether it represents a continuation of pre-incarceration drug use, or whether prison is also a setting for drug use initiation. This paper aims to describe drug use among people in prison in Norway and investigate risk factors associated with in-prison drug use.

**Methods:**

We used data from the Norwegian Offender Mental Health and Addiction (NorMA) Study, a cross-sectional survey of 1499 individuals in Norwegian prisons. Respondents reported on drug use (narcotics and non-prescribed medications) both before and during imprisonment. We used multivariate logistic regression to investigate the associations between drug use in prison and demographics, previous drug use, mental health, and criminal activity.

**Results:**

Sixty-five percent of respondents reported lifetime drug use, and about 50% reported daily use of drugs during the 6 months before incarceration. Thirty-five percent reported ever using drugs in prison, but initiation of drug used during incarceration was uncommon. In a multivariate model, factors independently associated with drug use in prison included lifetime number of drugs used (adjusted odds ratio [aOR] = 1.17; 95% confidence interval [CI] 1.12–1.23; *p* <  0.001), daily drug use in the 6 months before imprisonment (aOR = 7.12; 95%CI 3.99–12.70; *p* <  0.001), and being intoxicated while committing the crime related to current imprisonment (aOR = 2.13; 95%CI 1.13–4.03; *p* = 0.020).

**Conclusions:**

In-prison drug use is independently associated with high-risk drug use before imprisonment. To reduce drug use in prison, correctional services must systematically screen for pre-prison drug use and offer effective drug treatment for those in need.

## Background

The prison population worldwide is approaching 11 million people and continues to grow in the majority of countries (Walmsley, 2016). Among people in prison, a large proportion have a history of drug use and substance use disorders (SUD) (Fazel et al., 2017; Friestad & Kjelsberg, 2009; Stewart, 2009; UNODC, 2019). A recent systematic review and meta-analysis found that the pooled prevalence estimate for SUD was 51% among women and 30% among men (Fazel et al., 2017). People with SUDs in prison tend to have more wide-ranging mental and social problems including lower educational qualifications, lower rates of employment, more housing difficulties, poorer physical health, and more behavioral, psychological and psychiatric problems, compared to other inmates (Dolan et al., 2018; Kinner & Rich, 2018).

Drug use among people in prison is associated with a range of adverse outcomes both during imprisonment and post-release (Binswanger et al., 2013; Chang et al., 2015). The risk of suicide in prison is particularly high for people with SUDs, and withdrawal from drug use has been identified as a possible trigger for suicide in the first days of incarceration (Larney et al., 2014; Rivlin et al., 2013). In addition, people released from prison are at increased risk of death, especially from drug overdoses and accidents, and this risk is typically highest in the weeks immediately following release (I. A Binswanger et al., 2007; Bukten et al., 2017; Merrall et al., 2010).

Worldwide, about one in three people held in prisons is estimated to have used drugs at least once while incarcerated (UNODC, 2019). The European Monitoring Centre for Drugs and Drug Addiction (EMCDDA) has estimated that between 2% and 31% of people in European prisons inject drugs (EMCDDA, 2012). People who inject drugs are at increased risk for communicable diseases including HIV and hepatitis (Kinner et al., 2012), as sharing of needles occurs more frequently in prison than in community settings (van der Meulen, 2017). Previous research has also established that injecting drug use (IDU) during imprisonment is a strong risk factor for resuming IDU following release (Winter et al., 2016), and that IDU is an independent risk factor for re-incarceration after release from prison (Winter et al., 2019).

Although many people are incarcerated for reasons related to their drug use (Csete et al., 2016), for some, drug use may be initiated in prison. Some studies have found that drug use in prison follows drug use and SUD before prison, and that patterns of drug use in prison are a reflection of patterns of drug use before imprisonment (Cope, 2000; Strang et al., 2006). However, the literature on this issue is inconsistent, and it has been argued that the high-risk environment of prisons is particularly conducive to drug use initiation (Boys et al., 2002). Understanding the extent to which drug use in prison is a continuation of problematic drug use before imprisonment, or a new phenomenon initiated in custody, is essential to inform both prevention and treatment responses in custodial settings.

Prisons provide important opportunities for health interventions: a high proportion of people in prison have untreated SUDs and, in prison, they are more accessible for a period of time. According to the EMCDDA’s recent guide to health and social responses to drug problems, drug-related interventions in prisons and the correctional services may have significant impacts on morbidity, mortality and public health (EMCDDA, 2017). Research has shown that provision of opioid agonist treatment (OAT) during imprisonment is associated with a range of positive outcomes, including reductions in prison drug injection (Kinner et al., 2013), reduced risk of re-incarceration (Larney et al., 2012), and reduced risk of death after release from prison (Degenhardt et al., 2014). The detection of mental health problems and SUDs among people entering prisons, accompanied by evidence-based treatment and harm reduction measures, has the potential to improve both the health of people who experience incarceration, and the health of the communities to which they return.

From a public health perspective, time in prison could represent a turning point (EMCDDA, 2012, 2017). For that to happen, we need a better understanding of the trajectories into drug use in prison. In examining a large sample of adults incarcerated in Norway, the aims of this study were to (a) describe the patterns of drug use before and during imprisonment, and (b) identify factors associated with drug use in prison.

### Setting

Norway is a small, high-income country with approximately 5.5 million residents. The Norwegian criminal justice system is often described according to the “Scandinavian exceptionalism” characterized by low imprisonment rates and a comparably high level of care and services (Pratt, 2008, J. J. T. B. j. o. c, 2008). The incarceration rate in Norway was 73 per 100,000 persons in 2016, which is low compared to many other countries (e.g. 655 in the United States, 468 in Russia, 163 in Mexico, 114 in Canada) (WPB, 2019). In Norway, prison beds are spread across the country, to allow most prisoners to preserve geographical closeness to friends and family during prison (Bukten et al., 2015). All prisons are publicly funded and are categorized into high-security (almost two-thirds of prisons), low-security, or transitional housing units. The largest prison has a capacity of 400 people, while the smallest has only 15. Inmates often begin serving their sentences in high-security prisons before being transferred to a prison with lower security and later on to a transitional housing unit.

The longest sentence in Norway is 21 years of imprisonment. Release after two-thirds of one’s sentence is common. Of all releases from prison during 2018, about 20% of the inmates were released after 30 days or less and about 85% were released within 1 year (Kriminalomsorgen, 2018). Women constitute a minority in Norwegian prisons, with an annual proportion of about 6%.

The traditional Norwegian welfare model is comprehensive, institutionalized, and universal (Esping-Andersen, 1987), and, in contrast to many other countries, the Norwegian welfare state includes people in prison as part of its remit. The criminal justice system has rehabilitation and positive change as its main goals (Jewkes, 2015 and B; Bennett, J., 2015), and the Norwegian Correctional Services imports services such as health care, education, and cultural and social services from the public welfare system. People in prison with opioid use disorders may therefore both continue and initiate opioid agonist treatment while in prison and one can also apply to serve part of the prison sentence in residential facilities outside of prison. In addition, the Norwegian Correctional Services have both implemented drug treatment programs (Granheim et al., 2010) and separate SUD treatment units in prisons to achieve the goals of rehabilitation. However, the capacity of drug treatment is limited, as only 18 prisons have specialized SUD treatment units. Treatment shall ideally continue in community treatment settings upon release.

## Methods

### Participants

We based this cross-sectional study on self-reported data from the Norwegian Offender Mental Health and Addiction (NorMA) study (Bukten et al., 2015). The NorMA study is a large-scale study that uses national survey data to understand mental health, substance use, and criminal activity before incarceration among people in prison in Norway. All people imprisoned in Norway at the time of data collection were eligible to participate, irrespective of nationality, age, gender, or health status.

Researchers distributed and collected questionnaires to 57 of the 63 prison units in Norway, including high- and low-security units and transitional housing units, from June 2013–June 2014. Altogether 1499 individuals (98 women) responded to the questionnaire. The six units not included housed a maximum of 179 inmates and their non-participation was due to geographical inconvenience and limited researcher capacity. During 2014 the average prison population was 3717, and our sample thus includes approximately 40% of the population (Kriminalomsorgen, 2015). A full description of the study methods (Bukten et al., 2015) and previous publications based on the NorMA study have been published earlier (Muller & Bukten, 2019; Muller et al., 2018; Rognli et al., 2017).

### Procedure

#### Ethics

The NoRMA study was approved by the Norwegian Committee of Research Ethics (REK 2012/297), by the Ministry of Justice and Public Security, and by the Directorate of the Norwegian Correctional Services. Participation in the study was voluntary and based on written informed consent. Participants were informed that their answers were confidential from prison staff.

#### Instruments

The NorMA questionnaire included 116 questions related to drug use, mental and physical health, and criminal activity, and took approximately 30 to 60 min to complete. The questionnaire was available in Norwegian, English, Russian, French, and German, upon recommendation of the Norwegian Correctional Services.

##### Sociodemographic factors

Measures of sociodemographic variables included age, history of family problems, country of birth, education, and labor participation. History of family problems was indicated by growing up in a family with substance use or mental health problems. Country of birth was defined as born in a Nordic country or not, and education was defined as having completed secondary school or not. Persons who reported that they worked or studied prior to incarceration were defined as labour participants.

##### Mental health

Current psychological distress was measured by the 10-item Hopkins Symptom Checklist (HSCL10) with each item scored on a 1–4 scale (Derogatis et al., 1974). Mean item scores ≥1.85 were considered indicative of clinical concern (Strand et al., 2003).

##### Crime

Participants reported the types of crime related to their current incarceration. Many were charged and/or convicted for several types of crime; therefore, the types of crimes listed are not mutually exclusive.

##### Drug use

We asked participants about: lifetime drug use, drug use six-months before imprisonment, during former imprisonment, and during current imprisonment. We listed 16 different types of drugs, including non-prescribed use of medications such as benzodiazepines, methadone, and morphine. We defined individuals as having used any drug when responding positively to one or more drug type during each period. Lifetime use was defined as having used any drug (except alcohol) during any period. We also asked inmates whether they were under the influence of drugs or alcohol when they committed the crime(s) related to their current imprisonment.

Participants also provided information about their substance use during the 12 months before incarceration, by responding to the Alcohol Use Disorders Identification Test (AUDIT) (Saunders et al., 1993) and the Drug Use Disorders Identification Test (DUDIT) (Berman et al., 2005). Those who indicated through other questions that they had no experience with alcohol, non-prescribed medications or illicit substances were instructed to skip all other questions related to drug use. Consistent with standard cut-offs, harmful alcohol use was indicated by a score of ≥8 for men and ≥ 6 for women on the AUDIT (Babor, Higgins-Biddle, Saunders, & Monteiro, 2001; ROP, 2019). Harmful drug use was indicated by a score of ≥6 for men and ≥ 2 for women on the DUDIT (Berman et al., 2007). Reporting harmful alcohol use or harmful drug use was classified as “harmful substance use”. Drug use during current imprisonment was defined as the outcome.

### Statistical analyses

Descriptive statistics and regression models were estimated using SPSS version 25 and R version 3.6.0. A logistic regression model was fitted to estimate factors associated with the outcome, drug use during current imprisonment. Both univariate models for all covariates and a full, multivariate model were fitted. The coefficients were interpreted as odds ratios (OR) with 95% confidence intervals (95%CI).

### Missing data

In all 1499 questionnaires were available for analysis, of which 880 (58.7%) were completed for all relevant variables. The percentage of missing values ranged from 0 to 30.3%. A detailed list of missing values is given in Table 1. To reduce the potential bias of missing data we used multiple imputation (MI) to pre-process the data (Schafer & Graham, 2002). MI was performed using Multivariate Imputation by Chained Equations (MICE), which is a flexible tool for handling both continuous and categorical data under the missing at random (MAR) assumption (Buuren & Groothuis-Oudshoorn, 2011). Auxiliary variables were included to strengthen the MAR assumption (Van Buuren et al., 2006). Ten imputations were performed and combined using Rubin’s rule: estimated parameters from all complete data sets are pooled, giving a measurement of variation within and between the imputed data sets. MICE was employed using the function mice, and the prediction matrix was automatically generated using the quickpred function in the R package mice (Buuren & Groothuis-Oudshoorn, 2011). A sensitivity analysis was performed by comparing the results of regression models fitted to the data at hand (complete case analysis) and the data pre-processed with MI. The statistical significance of the covariates was not affected by the strategy for handling missing data. However, performing complete case analysis would mean deleting 41.3% of the available cases, which may result in unnaturally wide confidence intervals and potentially biased estimates. We therefore report the results of the analysis on the imputed data.

**Table 1:** Demographic characteristics, mental health, crime and substance use by gender (*n* = 1499)

	Men^**a**^ (***n*** = 1396)	Women^**a**^ (***n*** = 96)	Total (n = 1499)	Missing values, n (%)
**Sociodemographic factors**				
Age^b^, mean (SD)	35 (11.3)	35 (10.5)	35 (11.3)	131 (8.7)
History of family problems, n (%)	407 (29.2)	47 (49.0)	457 (30.5)	61 (4.1)
Non-Nordic borne, n (%)	385 (27.6)	15 (15.6)	404 (27.0)	49 (3.3)
Secondary school or more, n (%)	866 (62.0)	48 (50.0)	919 (61.3)	23 (1.5)
Work or study before incarceration, n (%)	688 (49.3)	36 (37.5)	721 (48.1)	50 (3.3)
**Current mental health**				
HSCL10 score^b^mean (SD)	1.9 (0.8)	2.1 (0.8)	1.9 (0.8)	320 (21.3)
**Crime**				
Crime related to current incarceration				88 (5.9)
Acquisitive crime, n (%)	420 (30.1)	26 (27.1)	450 (30.0)	–
Drug crime, n (%)	573 (41.0)	33 (34.4)	609 (40.6)	–
Violence, n (%)	574 (41.1)	31 (32.3)	606 (40.4)	–
Driving under the influence, n (%)	229 (16.4)	14 (14.6)	243 (16.2)	–
**Drug use measures**				
Number of drugs used in lifetime^b,c^, mean (SD)	5.4 (5.6)	5.0 (5.2)	5.4 (5.6)	58 (3.9)
Injection drug use in lifetime, n (%)	401 (28.7)	29 (30.2)	431 (28.8)	454 (30.3)
Daily use of drugs before prison, n (%)	656 (47.0)	43 (44.8)	702 (46.8)	163 (10.9)
Harmful substance use pre-incarceration, n (%)	876 (62.8)	57 (59.4)	933 (62.2)	35 (2.3)
Intoxicated when committing the crime, n (%)	885 (63.4)	60 (62.5)	947 (63.2)	127 (8.5)

^a^Gender: 7 missing values

^b^Continuous variable

^c^Including non-prescribed medications

## Results

### Sample characteristics

The sample included 96 (6.4%) women, and the mean age of participants was 34.6 years (median 32 years) (Table 1). More women than men reported having grown up in a family having problems with drug use, mental illness, or both. More than half reported having completed secondary school or more (Table 1).

Thirty percent reported only a primary school education, and 8% did not achieve any school qualifications at all. Both genders reported high scores on the HSCL-10 (mean score 1.9 for men and 2.2 for women). The majority of participants reported that their current incarceration was related to drug crime (40%) or violence (41%).

Among lifetime users of drugs, most had used multiple drugs (mean 5.4). Sixty-three percent reported being intoxicated (drugs, alcohol, or both) when committing the crime related to the current incarceration, and according to AUDIT and DUDIT scores, 62% had harmful drug use in the 12 months before incarceration.

### Pre-prison drug use

Of the 1499 respondents, 973 (65.2%) reported lifetime use of drugs. The different types of drugs used are shown in Table 2. Poly-drug use was commonly reported, often involving several substances. Of lifetime users (*n* = 973); 46% had used five or more types of drugs, and 30% had used ten or more types.

**Table 2:** Prevalence of lifetime and 6 months pre-prison drug use by type of drug and gender (*n* = 1499)

	Lifetime^**a**^, n (%)			6 months pre-prison, n (%)		
Type of drug^**b**, c^	Men	Women	Total	Men	Women	Total
Cannabis	855 (61)	54 (56)	913 (61)	634 (45)	40 (42)	677 (45)
Cocaine	713 (51)	45 (47)	761 (51)	350 (25)	13 (14)	365 (25)
(Meth) Amphetamine	667 (48)	48 (50)	718 (48)	457 (33)	33 (34)	490 (33)
Benzodiazepines^c^	612 (44)	44 (46)	659 (44)	454 (33)	35 (36)	490 (33)
Ecstasy	605 (43)	40 (42)	648 (43)	153 (11)	10 (10)	163 (11)
GHB	461 (33)	33 (34)	496 (33)	211 (15)	15 (16)	226 (15)
LSD, PCP or Ketamine	403 (29)	22 (23)	426 (28)	98 (0.7)	6 (0.6)	104 (0.7)
Methylphenidate, e.g., Ritalin	394 (28)	29 (30)	425 (28)	101 (0.7)	3 (0.3)	104 (0.7)
Morphine	394 (28)	24 (25)	419 (28)	179 (13)	11 (0.11)	190 (13)
Methadone, Buprenorphine	390 (28)	25 (26)	416 (28)	199 (14)	17 (18)	217 (14)
Heroin	361 (26)	26 (27)	398 (26)	155 (11)	13 (14)	168 (11)
Anabolic steroids	346 (25)	4 (0.4)	350 (23)	119 (0.9)	1 (0.1)	120 (0.8)
Synthetic cannabis	320 (23)	18 (19)	338 (23)	112 (0.8)	6 (0.6)	119 (0.8)
Inhalants	307 (22)	20 (21)	328 (22)	79 (0.6)	3 (0.3)	82 (0.5)
Other drugs/medications	201 (14)	12 (13)	214 (14)	106 (0.8)	5 (0.5)	112 (0.7)

^a^526 persons reported never having used drug

^b^Drugs/groups are not mutually exclusive

^c^All medications listed are non-prescribed medications

During the 6 months before their current imprisonment, 807 participants (54%) reported any drug use, and 47% reported daily use of drugs. Cannabis was the most commonly used drug (45%), followed by amphetamines (33%), benzodiazepines (33%), and cocaine (25%). Heroin was used by 11% (*n* = 168) (Table 2).

### In-prison drug use

Just over one-third of participants (*n* = 531, 35%) had used some form of drugs during previous or current imprisonment (Table 3). Of those, 18 individuals (3%) reported initiating drug use in prison. In-prison drug use was less common among women (25%) than among men (43%). More participants reported drug use during a past episode of imprisonment (*n* = 449) than during their current incarceration (*n* = 351). The most commonly used drugs during both former and current imprisonment were cannabis, benzodiazepines, OMT-medications, and amphetamines (Table 3).

**Table 3:** Prevalence of drug use during former and current imprisonment by type of drug and gender (*n* = 1499)

	Previous imprisonment, n (%)			Current imprisonment, n (%)		
Type of drug^**a**^	Men	Women	Total	Men	Women	Total
Cannabis	381 (27)	12 (13)	396 (26)	230 (17)	6 (6)	238 (16)
Cocaine	63 (5)	8 (8)	63 (4)	26 (2)	–	26 (2)
(Meth) Amphetamine	180 (13)	3 (3)	188 (13)	65 (5)	4 (4)	69 (5)
Benzodiazepines	339 (24)	7 (7)	347 (24)	166 (12)	5 (5)	172 (12)
Ecstasy	29 (2)	–	29 (2)	9 (1)	–	9 (1)
GHB	46 (3)	2 (2)	48 (3)	19 (1)	1 (1)	20 (1)
LSD, PCP or Ketamine	13 (1)	–	13 (1)	7 (1)	–	7 (1)
Methylphenidate, e.g., Ritalin	85 (6)	–	85 (6)	39 (3)	–	39 (3)
Morphine	77 (6)	–	77 (5)	30 (2)	–	30 (2)
Methadone, Buprenorphine	212 (15)	7 (7)	219 (15)	163 (12)	7 (7)	170 (11)
Heroin	101 (7)	8 (8)	109 (7)	29 (2)	1 (1)	30 (2)
Anabolic steroids	57 (4)	–	57 (4)	17 (1)	–	17 (1)
Synthetic cannabis	66 (5)	–	66 (4)	43 (3)	–	43 (3)
Inhalants	26 (2)	1 (1)	27 (2)	11 (1)	–	11 (1)
Other drugs/medications	22 (2)	–	23 (2)	13 (1)	–	14 (1)

^a^Groups are not mutually exclusive

### Factors associated with in-prison drug use

Of those who had used drugs during the current imprisonment (n = 351), almost all (96%) also reported using drugs during the 6 months before imprisonment. After adjusting for sociodemographic factors and factors related to mental health and criminal activity, the only factors related to prior drug use were independently associated with in-prison drug use (Table 4): number of drugs used in lifetime (OR = 1.12; 95%CI 1.07–1.17; *p* <  0.001), daily drug use in the 6 months before imprisonment (OR = 7.12; 95%CI 3.99–12.70; *p* <  0.001), and being intoxicated when committing the crime related to current imprisonment (OR = 2.13; 95%CI 1.13–4.03; *p* = 0.020).

**Table 4:** Factors associated with drug use in prison (*n* = 1499). Unadjusted and adjusted odds ratios (OR) and 95% confidence intervals (CI) from logistic regression

	Unadjusted		Adjusted	
	OR (CI)	*P*-value	aOR (CI)	*P-*value
**Sociodemographic factors**				
Men	1.88 (1.08–3.27)	0.027	2.03 (1.06–3.9)	0.034
Age (continuous)	0.97 (0.96–0.99)	< 0.001	0.99 (0.97–1.01)	0.262
History of family problems	2.01 (1.54–2.62)	< 0.001	1.13 (0.80–1.60)	0.489
Non-Nordic borne	0.49 (0.33–0.73)	< 0.001	1.45 (0.85–2.49)	0.172
Secondary school or more	0.58 (0.45–0.74)	< 0.001	1.00 (0.71–1.39)	0.974
Work or study before incarceration	2.72 (2.10–3.53)	< 0.001	0.98 (0.69–1.38)	0.889
**Mental health status during prison**				
HSCL10 score (continuous)	1.03 (1.01–1.04)	0.002	1.00 (0.98–1.02)	0.947
**Crime**				
Crime related to current incarceration				
Acquisitive crime	2.37 (1.84–3.05)	< 0.001	1.26 (0.90–1.77)	0.177
Drug crime	3.05 (2.34–3.98)	< 0.001	1.00 (0.70–1.43)	0.993
Violence	0.95 (0.73–1.23)	0.670	0.93 (0.66–1.31)	0.673
Driving under the influence	2.91 (2.14–3.95)	< 0.001	1.23 (0.83–1.81)	0.299
**Drug use measures**^**a**^				
Number of drugs used in lifetime (continuous)	1.24 (1.21–1.28)	< 0.001	1.12 (1.07–1.17)	< 0.001
Daily use of (any) drugs before prison	18.61 (11.99–28.89)	< 0.001	7.12 (3.99–12.70)	< 0.001
Injection drug use in lifetime	5.16 (3.88–6.85)	< 0.001	1.21 (0.81–1.81)	0.356
Harmful drug use	8.57 (5.68–12.93)	< 0.001	0.73 (0.39–1.38)	0.329
Intoxicated when committing the crime	9.68 (5.92–15.82)	< 0.001	2.13 (1.13–4.03)	0.020

^a^Including non-prescribed medications

## Discussion

In this national survey study of 1499 adults imprisoned in Norway, almost one in two reported daily drug use in the 6 months before incarceration. However, very few (3%) reported initiating drug use in prison. After adjusting for covariates, we found that prior drug use was strongly associated with drug use in prison: people who had used more drug types in their lifetime, those who reported daily drug use in the 6 months before imprisonment, and those who reported being intoxicated when committing the crime related to their current imprisonment, were more likely to also report drug use in prison.

Consistent with previous research in Europe and elsewhere (Fazel et al., 2006; Fazel et al., 2017; Friestad & Kjelsberg, 2009; Rowell-Cunsolo et al., 2016; Stewart, 2009; Strang et al., 2006), the majority of participants in this study reported a lifetime history of drug use, and many had used multiple substances.

As lifetime use capture both single use and substance use disorders, daily use in the 6 months before prison is likely a better indicator of harmful use. Our study revealed that almost 50% of inmates used drugs on a daily basis prior to incarceration, with a somewhat lower prevalence among women (45%). The overall prevalence is in line with the literature, although a recent meta-analysis conducted by Fazel and colleagues found the opposite gender pattern: 51 % of women, compared with 30% of men were estimated to have suffered from drug use disorders during the year before their incarceration (Fazel et al., 2017).

Cannabis was the most commonly used drug; cocaine and amphetamines were both used by approximately half; and around one in four had used heroin. This is in line with international studies reporting that cannabis is the most commonly used illicit drug among people who experience incarceration, with between 14% and 70% having used it at some time in their lives (Carpentier et al., 2018). Whereas a small proportion of the general population has ever used heroin (EMCDDA, 2019), the lifetime prevalence among people in prison is typically much higher, with prevalence rates between 4% and 37% in European countries (Carpentier et al., 2018). Our findings add to a substantial body of literature highlighting the need for evidence-based drug treatment in prison settings, at a scale proportionate to need.

Although drug use was reported in prison, fewer people used drugs in prison than before imprisoned. In our study, around one in three participants reported drug use during any imprisonment (either current or former), and almost one in four reported drug use during their current imprisonment. These findings are in line the Carpentiers and colleagues’ recent review of 59 studies of drug use in European prisons (Carpentier et al., 2018), finding that drug use continues to some extent within the prison setting. The authors note that there are large variations between studies, but the proportion of inmates having used drugs in prison lies between 20% to 45% in most studies (Carpentier et al., 2018). Again consistent with previous research (Rowell-Cunsolo et al., 2016; UNODC, 2019), the drug most commonly used in prison was cannabis. Given the very high rates of drug use before incarceration, it is perhaps unsurprising that, despite considerable reduction in supply, some continue to use drugs in prison. In tandem with prison-based drug treatment, there is a clear need for harm reduction services in prison settings to mitigate the harms associated with drug use, particularly the injection of cocaine, amphetamines, and heroin.

A secondary aim of our study was to consider the extent to which drug use is initiated in prison. In our study, those who reported using drugs in prison were not first-time users: almost all reported having used drugs in the 6 months before imprisonment. Only 3% of those who reported in-prison drug use also reported having initiated drug use during prior or current imprisonment. This finding is also consistent with previous research: a recent review found that between 0.5% and 10% of inmates report initiating drug use in custody (Carpentier et al., 2018).

After adjusting for covariates, pre-incarceration drug use was a strong predictor of drug use in prison. This is not surprising as those engaging in daily drug use before incarceration likely experienced withdrawal or craving during imprisonment. Earlier studies have found that inmates who were dependent on heroin or cocaine before incarceration were more likely to use heroin in prison (Cope, 2000; Rowell-Cunsolo et al., 2016) and that heroin and cocaine users were more likely to have used drugs during imprisonment (Rowell-Cunsolo et al., 2016). Our results thus support previous studies suggesting that in-prison drug use is typically a continuation of pre-incarceration drug use behavior (Cope, 2000).

### Strengths and limitations

Conducting research on drug use in prison is complex, and heterogeneity in research design, sampling, response rate, and measurement make meaningful comparisons between studies difficult (Carpentier et al., 2018). Self-report data on drug use before and during imprisonment may have several limitations concerning validity and reliability. First, respondents may have had difficulty recalling information about lifetime drug use and criminal activity, leading to under-estimations. In addition, in-prison drug use is likely to be under-reported, given that disclosures may lead to sanctions or penalties (Carpentier et al., 2018). Participants who have been incarcerated for more extended periods may be particularly reluctant to report current drug use because they are close to being released, and may be concerned that disclosures could jeopardize their freedom (Rowell-Cunsolo et al., 2016).

To overcome some of the limitations associated with previous studies, the NorMA study covered most prison units in Norway. Although our sample (*n* = 1499) was not drawn randomly from the official prison population, it was found to be representative of the national prison population in terms of gender, citizenship, and country of birth (Bukten et al., 2015). To mitigate concern that disclosure of drug use would not be confidential, study investigators personally administered data collection at all stages, both related to information, distribution and collecting questionnaires.

## Conclusions

In our study, people in Norwegian prisons reported a high lifetime prevalence of pre-prison drug use. Those who reported using drugs in prison were characterized by high-risk drug use before prison, involving poly-drug use and daily drug use – a group of people in apparent need for treatment. Our findings illustrate the importance of developing prison-based programs to address substance use problems and highlight the potential for effective drug treatment in the community.

A consistent challenge in public health is to provide services to the people who need them the most and are hard to reach. From a public health perspective, time in custody could therefore represent a rare opportunity to identify individuals with a history of harmful substance use, and initiate SUD treatment among a highly disadvantaged segment of the community. In line with the United Nations’ Standard Minimum Rules for the Treatment of Prisoners, the Nelson Mandela Rules, (UN, 2016), key to realizing this opportunity is routine screening for substance use at prison reception, provision of evidence-based drug treatment and harm reduction services in prison, at a scale proportionate to need, and mechanisms to promote continuity of treatment as these individuals transition back to the community.

Advancing our knowledge of traditionally marginalized and understudied groups, such as people with SUD in prison, is important to understanding social disparities in health. Responding to the SUD treatment needs of people who experience incarceration is an essential part of broader efforts to reduce health inequalities, in Norway and globally.

## Data Availability

The data that support the findings of this study are available on reasonable request from the corresponding author AB. The data are not publicly available due to them containing information that could compromise research participant privacy and consent.
